# JUICE: a data management system that facilitates the analysis of large volumes of information in an EST project workflow

**DOI:** 10.1186/1471-2105-7-513

**Published:** 2006-11-23

**Authors:** Mariano Latorre, Herman Silva, Juan Saba, Carito Guziolowski, Paula Vizoso, Veronica Martinez, Jonathan Maldonado, Andrea Morales, Rodrigo Caroca, Veronica Cambiazo, Reinaldo Campos-Vargas, Mauricio Gonzalez, Ariel Orellana, Julio Retamales, Lee A Meisel

**Affiliations:** 1 Millennium Nucleus in Plant Cell Biology and Plant Biotechnology Center, Andres Bello University, Santiago, Chile; 2 Laboratorio de Genética Molecular Vegetal, Departamento de Biología, Facultad de Ciencias, Universidad de Chile, Santiago, Chile; 3 Laboratorio de Bioinformática y Expresión Génica, INTA, Universidad de Chile, Santiago, Chile; 4 INIA La Platina, Santiago, Chile; 5 Facultad de Ciencias Agronómicas, Universidad de Chile, Santiago, Chile; 6 Valent BioSciences Corporation, Santiago, Chile

## Abstract

**Background:**

Expressed sequence tag (EST) analyses provide a rapid and economical means to identify candidate genes that may be involved in a particular biological process. These ESTs are useful in many Functional Genomics studies. However, the large quantity and complexity of the data generated during an EST sequencing project can make the analysis of this information a daunting task.

**Results:**

In an attempt to make this task friendlier, we have developed JUICE, an open source data management system (Apache + PHP + MySQL on Linux), which enables the user to easily upload, organize, visualize and search the different types of data generated in an EST project pipeline. In contrast to other systems, the JUICE data management system allows a branched pipeline to be established, modified and expanded, during the course of an EST project.

The web interfaces and tools in JUICE enable the users to visualize the information in a graphical, user-friendly manner. The user may browse or search for sequences and/or sequence information within all the branches of the pipeline. The user can search using terms associated with the sequence name, annotation or other characteristics stored in JUICE and associated with sequences or sequence groups. Groups of sequences can be created by the user, stored in a clipboard and/or downloaded for further analyses.

Different user profiles restrict the access of each user depending upon their role in the project. The user may have access exclusively to visualize sequence information, access to annotate sequences and sequence information, or administrative access.

**Conclusion:**

JUICE is an open source data management system that has been developed to aid users in organizing and analyzing the large amount of data generated in an EST Project workflow. JUICE has been used in one of the first functional genomics projects in Chile, entitled "Functional Genomics in nectarines: Platform to potentiate the competitiveness of Chile in fruit exportation". However, due to its ability to organize and visualize data from external pipelines, JUICE is a flexible data management system that should be useful for other EST/Genome projects. The JUICE data management system is released under the Open Source GNU Lesser General Public License (LGPL). JUICE may be downloaded from  or .

## Background

Within the last 10 years, there has been an exponential growth in the number of genomes that have been completely sequenced [1-4]. This rapid release of large quantities of data has made it necessary that high-throughput annotation and data management systems be developed, such that the information may be accessed and analyzed reliably, accurately and efficiently.

The sequencing, assembly and annotation of complete eukaryotic genomes are costly and time consuming. For this reason, numerous EST projects are underway to identify candidate genes associated with specific biological processes [5-9]. EST sequencing projects set the platform for functional genomics analyses using microarrays and digital expression analyses [10,11]. Additionally, since ESTs are fragments of sequences from cDNAs, it is possible to identify putative functions of these gene fragments by bioinformatic analyses such as similarity based searches [12-14].

Despite the obvious advantages associated with an EST Project, the EST Project Workflow creates a large quantity of data that must be processed, stored and analyzed. EST sequence data is usually examined through a number of different bioinformatic tools such as assembly tools (i.e. Phrap [15-17] or CAP3 [18]), similarity search software (i.e. BLAST [19]), filters scripts, and sequence analysis programs (i.e. InterProScan [20]), among others. A large number of EST projects [21-25] use these types of tools, differing only in the software, filters and/or parameters used or the order in which these tools are applied [26-28].

Within the same EST project, it may, at times, be necessary to use these tools in a flexible manner such that more accurate data may be obtained. For example, by altering the parameters in programs such as Phrap or CAP3, investigators vary the stringency with which they are assembling the Contigs, thereby identifying closely homologous gene families, altered poly-A tails, alternative splicing, SNPs, etc [29-32]. Additionally, annotations associated with sequences may change as more information is available. For example, a sequence which is annotated as "unknown function" may be assigned a function in the future based on the increasing sequence information that is publicly available.

Due to the size and complexity of the results that are obtained from using each of these tools, the development of data management systems that give easy and organized access to these results is critical. It is also very important that the system provides clear information about the processes and parameters that have been applied to the data.

Most of the existing systems that handle EST project data are designed to represent a particular project environment with a linear analysis of data (a linear pipeline) and are developed to work with specific bioinformatic tools [22,24,25,33-37]. These software programs normally have user interfaces that are adapted to solve a specific problem and do not enable the users to add extensions or compare parallel processes (a branched pipeline), thus limiting their flexibility and application to new sequence analyses, new EST projects and/or the future development of the software. Due to the increasing number of bioinformatic tools available to analyze sequence data, it is important that an EST Data management system be developed such that the system will be able to receive and search general pipeline processes and data of an EST Project Workflow, while simultaneously be easily adapted to work with new specific requirements and bioinformatic tools.

To meet these requirements, we have developed JUICE, an open source data management system that is able to receive and search general pipeline processes and data from an EST Project Workflow such as that which we developed within the framework of one of the first functional genomics projects in Chile, entitled "Functional Genomics in nectarines: Platform to potentiate the competitiveness of Chile in fruit exportation" [38-40]. In comparison to other systems, the JUICE data management system does not require a well defined linear pipeline [22,35]. Rather, JUICE allows a branched pipeline to be established, and modified, during the course of an EST project. This branched pipeline enables the user to compare results between two or more parallel processes (i.e. between two distinct filters or two distinct assembly processes). Additionally, it enables the users to visualize the history of the processes and results. JUICE lets the user compare the results of multiple processes, with a search engine that searches internally within all the branches of the pipeline. There is a clipboard included in JUICE, which enables the user to select specific data sets to be use as inputs in other processes, to be stored into new working groups or to be downloaded. These characteristics of JUICE make it a dynamic and flexible tool for storing and accessing large datasets in an efficient and user-friendly manner.

## Implementation

### JUICE model

**Figure 1:**
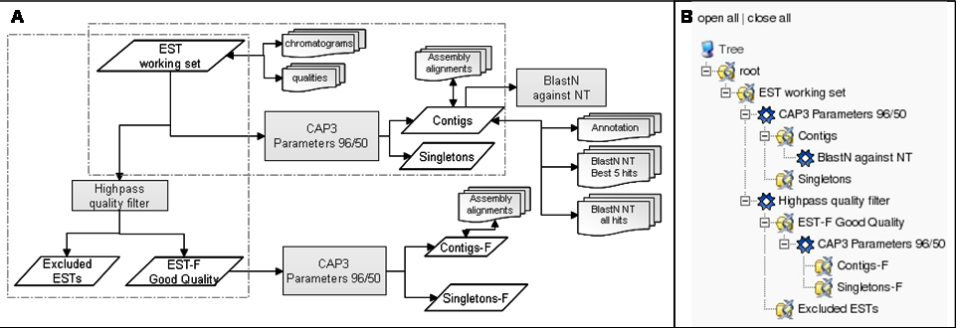
**Module-based modeling of JUICE permits a flexible branch pipeline of information associated with an EST Project workflow to be organized and accessed**. This figure represents an EST project workflow that has been model into JUICE. A) An EST project workflow organized into modules. B) A tree representation of this EST project workflow as it appears in the JUICE web interface. The EST project workflow in panel A has been organized into modules. Two examples of modules have been marked by the light gray dashed boxes. Each module incorporates a group, process and the products of the process. In the example in the horizontal dashed box, the group would be the "EST working set"; the process would be "CAP3 parameters 96/50"; and the products of the process would be "Contigs" and "Singletons". Similarly in the example in the vertical dashed box, the group would be "EST working set"; the process would be a "Highpass quality filter"; and the products of the process would be the "Excluded ESTs" as well as the "ESTs that are good quality". Because of this module-based modeling of JUICE, additional modules may be added, deleted and/or modified without the need to create a new pipeline. Additionally, searches may be performed between the different branches of this pipeline. Rectangles represent processes; rhomboids represent sequences that may be groups and/or products of processes; multidocument forms represent additional information that may be associated with the sequence information (i.e. quality information, chromatograms, annotations, etc.). NT: Non-redundant (NR) database in NCBI. EST-F: ESTs that passed the filter process. Contigs-F: Contigs formed in the assembly process using the ESTs that passed the filter process as the input. Singletons-F: Singletons formed in the assembly process using the ESTs that passed the filter process as the input.

JUICE has been modeled such that it may form a flexible branched pipeline. This branched pipeline is possible because JUICE is modeled based upon modules. Each module contains a *group*, *process *and the *products *of the process. By modeling JUICE in this way, modules may be added at any time in the EST Project Workflow. These modules may be organized into a multiple branched pipeline, thereby increasing the flexibility of this data management system (Figure 1).

Groups are inputs such as groups of sequences in FASTA format. The processes are descriptions of processes that have been performed on these sequences groups (NOTE: JUICE does not run the processes. Rather, JUICE provides the user with a very easy and efficient method to upload and organize the output results from external processes into a database.) The products are the results (outputs) of a process. The input for every process is a sequence group and the product can be as many sequence groups as the process needs to generate. The process, once applied, can provide extra information about both the sequence group and the products. The products of a process may also serve as a group (or input) of other processes.

An example of a multiple branched pipeline that is generated from an EST Project Workflow and modeled in JUICE is shown in Figure 1. In this example, an assembly tool takes a number of sequences and their quality information as inputs and generates two groups of sequences, contigs and singletons. Alternatively, filters such as Vector Masking [41] and Trimmer X [42] may take a group of sequences with associated chromatograms, quality values, characteristics and/or annotations, and modify/eliminate some of the sequences within these groups [43]. As mentioned earlier, to simplify this analysis and model a branched pipeline with this information, we have defined *groups*, *processes *and *products*, in order to abstractly represent EST project workflows. The numbers and types of groups, processes and products may vary depending upon the needs of a particular project.

### System architecture

**Figure 2:**
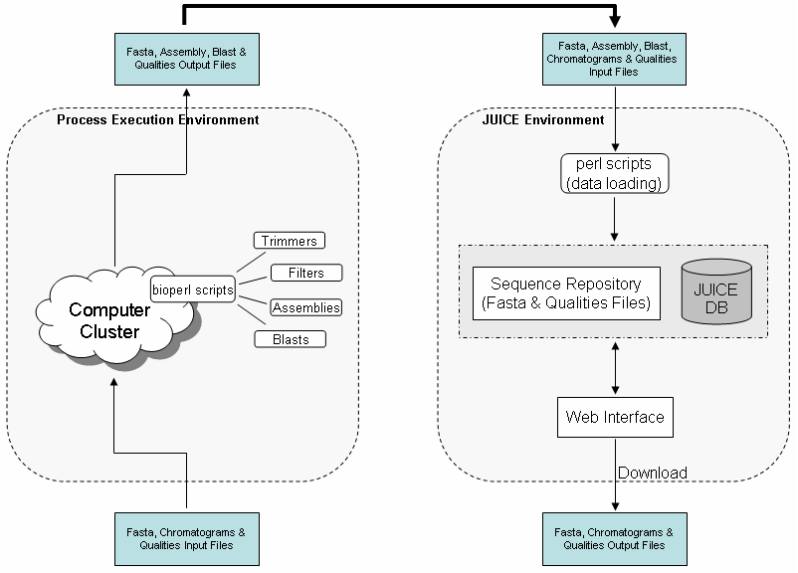
**The architecture of JUICE has been designed based upon two environments: process execution and JUICE**. The process execution environment contains the processes and different bioinformatics tools used to analyze sequences in FASTA format, sequence chromatograms as well as quality input files. The results or outputs of the process execution environment are the input files that are used by JUICE. Input FASTA or qualities files are loaded, using Perl scripts, into the JUICE database. Information can be visualized in the JUICE Web Interface and downloaded for performing additional filters and/or processes.

The JUICE architecture is illustrated in Figure 2. This figure shows not only the architecture of the JUICE environment (right side) but also illustrates how JUICE can be associated with a process execution environment, taking the output of bioinformatic processes, loading them into the database and allowing the user to work with these outputs. Information loaded into the JUICE database can be downloaded from JUICE and used in any way the research team deems necessary. The processed data can, then, be loaded back into JUICE and reflect the evolution of the data analyses.

Figure 2 is an example of our own project environment [38-40], in which we use a cluster of several computers to run bioinformatic processes (left side). The outputs of our project environment are usually FASTA files [44], qualities files or chromatograms. These output files are loaded into JUICE.

### JUICE platform

JUICE may be installed in a web server with PHP4 [45] or later versions and a MySQL 5 [46] database. Perl scripts are used to load data into the database. JUICE has been developed primarily using object oriented techniques and the use of templates for the Graphical User Interface (GUI). A Java Applet has been used for sequence chromatogram visualization [47].

Much of the information in JUICE may be stored in files that are external to the JUICE data management system. Sequence information may be stored in FASTA [42] files that are indexed by JUICE and are used as an external resource whenever this information needs to be displayed. Testing JUICE with greater than 400,000 sequences and associated data has demonstrated that JUICE performs efficiently and accurately [38-40].

## Results

### JUICE is a workflow independent web platform for EST analyses

JUICE provides the user with a very easy and efficient method to organize and visualize output information from many bioinformatics tools. The modular model of JUICE permits a branched pipeline to be formed. The complexity of this pipeline can increase as the project progresses. Additionally, since the bioinformatic tools are external to JUICE, the user can customize their pipelines without being limited to software design, as seen in other specific pipeline software [22,35].

### User-friendly features are incorporated into JUICE

**Figure 3:**
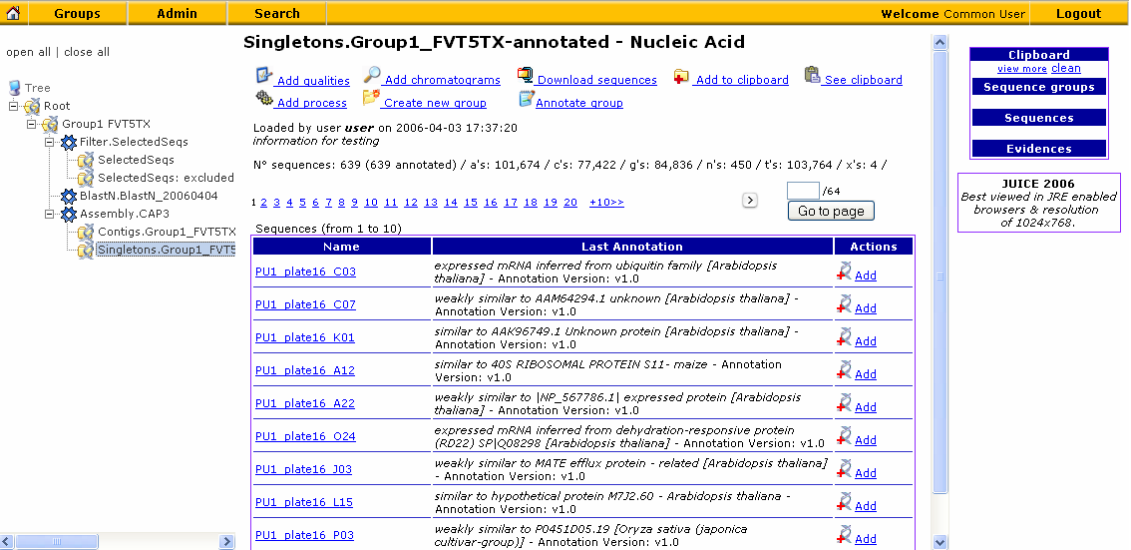
**Screenshot of the JUICE web interface**. The JUICE web interface enables the user to easily browse and/or search through the information that has been uploaded into the branched pipeline of JUICE. This figure demonstrates a screenshot of JUICE where the singletons of a group of sequences are shown. The position of this group of sequences within the branched pipeline may be viewed in the tree on the left-hand side of the image. The detailed information of this group may be seen in the center of the screen. When additional information is associated with this group this may be visualized on this screen. The example shown here, displays the annotation of each sequence. The user can select the name of each sequence to obtain more information (i.e. nucleotide sequence, quality information, chromatograms, if available). The search engine enables the user to find sequences by name, annotation or other associated information. The Clipboard, allows the user to select and store specific sequences for future analyses.

Several features have been added to JUICE that enable the user to easily install, upload, search and analyze the information that is stored in the JUICE data management system (Figure 3). The features available in the JUICE Data management system may be separated in three categories; *data loading*, *data visualization *and *administrative tools*. Below, each category is described in detail.

#### Data loading

The JUICE web interface provides an easy way to load output files from CAP3 [18], Phrap [15-17] or any output that is in a FASTA format. JUICE provides a web interface for loading BLAST [19] results by asking the user to indicate the location of a database with such results. The compatible formats are described in more detail in the JUICE User Manual.

The data loading features of JUICE include:

##### Create groups

A group can be created from a FASTA file, as a subset of sequences from another group, or an empty group that could be used as a folder simply for structuring information.

##### Load quality values into a sequence group

**Figure 4:**
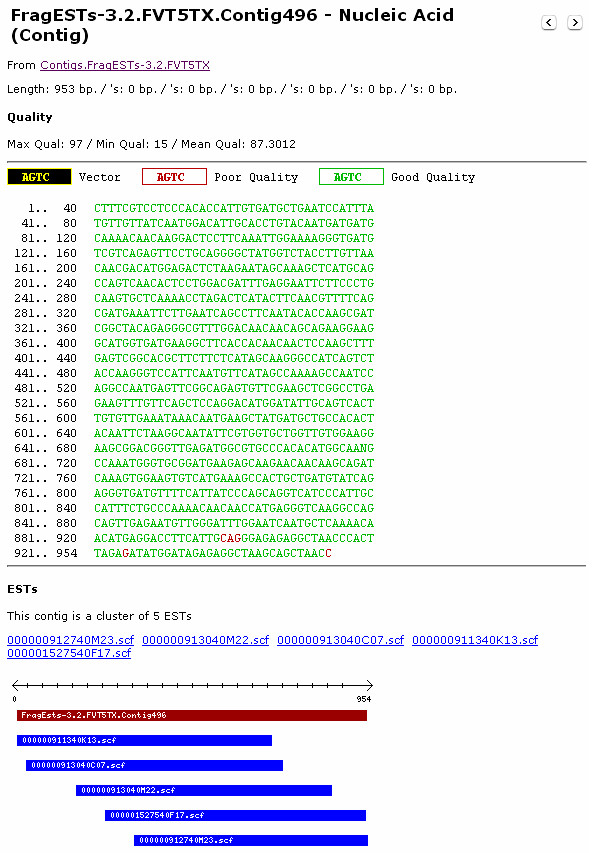
**Screenshot of Sequence Information using the JUICE web interface**. JUICE integrates all the information associated with a sequence (i.e. nucleotide sequence, quality information, chromatograms, etc). This figure demonstrates how JUICE displays the consensus sequence of a contig. The color of each nucleotide represents the quality of each base. Additionally, when contigs are visualized, the EST composition of the contig and the position of each EST within the consensus sequence of the contig are seen at the base of the screen. The user may then select each EST that forms a part of this contig, in order to visualize more detailed information about each EST.

Quality values associated with a sequence group can be loaded by providing JUICE with the quality files for that particular sequence group. Once quality information is loaded for a certain group, the user will be able to see the quality of the bases in these sequences by the color of the bases (Figure 4).

##### Load chromatograms into a sequence group

Chromatograms associated with a sequence group can be loaded into JUICE by specifying the directory in which the chromatogram files are stored. JUICE will automatically find chromatogram files by matching filenames from this directory with the sequence names from the group. Once chromatograms are loaded into a certain group, the option "See Chromatogram" will appear every time a sequence is displayed.

##### Upload results from a filter process

The results of a filter process can be uploaded into JUICE. An example of a filter process would be, for example, a script that takes a sequence group in FASTA format (with associated quality information for these sequences) and filters out all the sequences that are "poor quality". In this case, the input of the filter process would be all the sequences in the group. The output would be all the sequences that passed this filter, "good quality" sequences. [Note: the filter processes are run externally. The information (input and output) of these processes are stored and accessed by JUICE]. JUICE will create an output group with the sequences from the file containing the results. Additionally JUICE will create a second group containing the sequences that didn't pass the filter (the ones that are in the input group and do not appear in the output).

##### Upload results from an assembly process

The results of an assembly process that was applied to a group of sequences can be uploaded into JUICE. By providing JUICE with the result of an assembly program such as Phrap or CAP3 (*.ace output files), JUICE will display the results of this assembly in two groups: contigs and singletons. By navigating the singletons group, information associated with the original sequences can be visualized. Within the contig group, the user can visualize a digital image, representing the original sequences and their position in the assembled contig (Figure 4). By clicking on the name of the contig, the user may easily access the information associated with each sequence in this contig.

##### Upload BLAST results

The results of a BLAST search applied to a group of sequences can be uploaded into JUICE. By providing JUICE with the location of a database containing the results of a BLAST search, JUICE will permit the user to easily navigate between sequence information and the results of similarity searches.

#### Data visualization

The JUICE web interface enables the user to visualize the information in a graphical, user-friendly way. JUICE enables the user to **browse sequence groups **as if they were folders. As seen in Figures 1 and 3, each folder represents a sequence group and the navigation tree displays the processes applied to each group. By clicking a particular group, the user can visualize a paginated list of sequences. Summary data such as annotations and other details associated with the processes applied to the particular group are also displayed.

JUICE also enables the user to **visualize detailed information for each sequence**. This information includes annotations, color coded quality information associated with each base (if loaded) and the sequence itself (Figure 4). When viewing a contig, the EST composition of the contig and the position of each EST within the consensus sequence of the contig are visible. BLAST results, as well as other useful information associated with the sequence will be displayed, if available.

A **Clipboard **tool is also available in JUICE. The clipboard tool lets the user select a set of sequences, while he/she is browsing the groups, sending them to the Clipboard. At any time, the user can take the sequences that have been stored in the Clipboard and create a new group with these sequences or download them. This provides a way of generating a sort of "filing system", where groups are folders and sequences are files. This enables the researcher to organize the information that is most useful for a particular analysis.

JUICE has a powerful **Search Tool**. This tool enables the user to search throughout the branched pipeline. The user can easily and rapidly find sequences by specifying keywords that may be contained in sequence names, group names, annotations, etc. The user may send the search results to the clipboard such that a new group of sequences may be created or the results may be downloaded for further analyses.

#### Administrative tools

JUICE has been developed such that the user will have access privileges associated with their role. Access may be given as an **administrator **or as a **user**. The Administrator can create different user profiles with different levels of access to JUICE data. The administrator can modify a user's access to JUICE, allowing or denying the ability to view/edit sequence groups, post news and other administrative privileges.

A **news **section is available in JUICE. A user with access to news can post, edit, delete and hide news. The information added in the news section may be viewed by all JUICE users.

JUICE has been implemented with an option to visualize **different version **of information associated with a sequence, such as annotations. In this case, each sequence can have one current annotation (the one that is visible to the user) and a list of old annotations, which are not visible. Administrators can choose which versions are visible to other users.

## Discussion

EST projects and other genome oriented projects are continuously generating large amounts of information. The development of systems that centralizes and enables the information to be easily accessed, integrated and analyzed by all the users of the project increases the value of this information. We have developed JUICE, an open source data management system which enables information generated in genome projects to be easily and efficiently integrated, organized and accessed.

Many previously reported EST data management systems are designed to represent a particular project environment with a linear analysis of the data (a linear pipeline) and are developed to work with specific bioinformatic tools [22,24,25,33-37]. These software programs normally have limited user interfaces that are oriented towards solving a specific problem, thereby limiting the flexibility and application of the software to new sequence analyses, new project analyses or future software development.

In contrast to other data management systems, the JUICE data management system does not require a well defined linear pipeline. JUICE has been modeled so that it may form a flexible branched pipeline of modules. Each module contains groups (inputs), processes and products (outputs). Unlike other data management systems, the JUICE pipeline may be modified and/or expanded without the need to create a new pipeline [22,35]. JUICE's flexibility, therefore, allows the project workflow to be modified during the course of a project. It is not necessary to fully define the pipeline at the beginning of the project. The user can externally run a set of initially defined processes and load the corresponding inputs and outputs into JUICE. Then, based upon the analyses of the results, the user can decide what other processes need to be run. This creates a dynamic workflow in which the results of one process may be compared efficiently with the results of another.

Other pipeline generation systems such as EGene [48] also permit the development of branched pipelines that integrate the data generated in an EST project workflow. However, these other systems do not enable the user to alter, modify or extend the pipeline that has been created [48]. JUICE enables the user interact with the pipeline, not just visualize data. The user can modify the branches of the pipeline or extend the pipeline by creating new modules, using a user-friendly interface.

Additional features have been incorporated into JUICE making it easier for the users to access and search the information that is available in the multiple branches of the pipeline (results from different processes). The search engine enables the user to find sequences by name, annotation or other associated information. One of the benefits of this search engine is that it enables the user to compare the ESTs that form one Contig under specific assemble conditions, with the ESTs that form a distinct Contig under different assemble conditions. The Clipboard, allows the user to gather a set of manually selected sequences, such that this set can be downloaded in a compressed file (*.tar or *.gz format) or used to create a new group for future analyses. The results of these future analyses may then be uploaded in JUICE and associated with both the old and new groups that contain these sequences. The user-friendly web interfaces enable the user to easily browse through sequences, visualizing the bases of a sequence, quality information, contig composition, chromatograms and BLAST results. JUICE integrates all these features in a branched pipeline, whose structure may be visualized in the schematic tree that appears on the left-hand side of the JUICE interface. This tree may be expanded or compacted to show the details associated with each group, process and products of the process.

## Conclusion

In conclusion, we have developed JUICE, a unique user-friendly and flexible module-based, open source data management system that has been developed to aid users in organizing and analyzing the large amount of data generated in an EST Project workflow. In comparison to other data management systems, JUICE pipelines may be modified and/or expanded to meet the changing needs of EST projects as well as other genomics based projects. By using the web-based interface, users may easily browse or search the multiple branches of the pipeline in order to analyze and compare the results of various processes, as well as store selected information into a clipboard. By accessing the information generated from different bioinformatic analyses in a more user-friendly and graphic manner, JUICE may serve as a useful tool for comparative sequence analyses such that new information hidden in the sequences may be unveiled.

## Availability and requirements

JUICE has been tested successfully in server machines using Linux (Fedora Core 5, Ubuntu 6, Gentoo 2006, Suse 10.1 and Mandriva 2007) as the operating system with Apache, PHP, Perl and Mysql packages installed. In order to see the graphics, the GD library of PHP is needed. When navigating JUICE, the user will need a web browser, such as Internet Explorer or Firefox, with Java installed to see the chromatograms. JUICE has been released under the GNU Lesser General Public License (LGPL) and it can be downloaded from  or . A Readme file with installation instructions is available with the downloadable JUICE package. A user/administrator manual has been provided in order to aid in the installation and use of JUICE.

## Abbreviations

BLAST Basic Local Alignment Search Tool

EST Expressed Sequence Tag

GUI Graphical User Interface

LGPL Lesser General Public License

SNPs Small Nucleotide Polymorphisms

Perl Practical Extraction and Report Language

## Authors' contributions

ML designed the original functionalities of JUICE along with the design and development of database schemas and the initial system prototype. JS, CG and ML further developed and improved JUICE. PV, VM, JM, AM, and RC tested and critiqued JUICE as end-users. HS and LM conceived the system, participated in its design and coordination, and supervised the development and implementation of the JUICE software. ML, JS, HS and LM drafted the manuscript. HS, VC, RC, MG, AO, JR and LM supervised the Chilean Functional Genomics Consortium in Nectarines which provided the EST project workflow data which was utilized in the development and testing of JUICE. All authors read and approved the manuscript.
